# Evaluation of Popularity, Reliability, and Quality of Dental and Oral Microbiology Videos on YouTube as Sources of Dental Education

**DOI:** 10.1155/ijod/1509354

**Published:** 2026-02-24

**Authors:** Ahmed Hashim, Anil Bangalore Shivappa

**Affiliations:** ^1^ Department of Biomedical Sciences, College of Dentistry, King Faisal University, Al-Hassa, 31982, Saudi Arabia, kfu.edu.sa

**Keywords:** dental microbiology, oral microbiology, popularity, quality, reliability, YouTube

## Abstract

**Objectives:**

The expansion of teaching resources in dental education has changed significantly because of the growth of digital video platforms. However, questions remain about their reliability and quality. This study aims to evaluate the popularity, reliability, and quality of YouTube videos on dental and oral microbiology.

**Materials and Methods:**

Two search phrases were used to identify 200 videos, and the video power index (VPI) was utilized to evaluate popularity based on views, likes, and dislikes. The GQS scoring system was used to measure the videos’ quality, and JAMA and modified DISCERN tools were used to assess their reliability.

**Results:**

Analyzed videos were classified into four categories: poor, moderate, good, and excellent. VPI of the 61 videos analyzed was moderate (0.06), and JAMA and mDISCERN both achieved low excellent reliability ratings (9.8% and 3.3%), respectively. However, only 6.6% of the videos received a GQS quality rating of excellent.

**Conclusion:**

YouTube algorithms favor shorter content for student engagement and influence the video’s popularity. The absence of stringent standards for reliability and quality during production led to the conclusion that a significant proportion of videos was educationally ineffective. Thus, it is essential that educators carefully plan, write reliably, and produce high‐quality videos.

## 1. Introduction

The oral cavity is the gateway to the external environment and is colonized by bacterial, fungal, and viral microbiota. This dynamic microbial ecology affects oral homeostasis and influences dental and systemic disorders. Social media has facilitated online education during the COVID‐19 pandemic and transformed teaching by providing various options for knowledge sharing and student involvement. Recent research shows that many online educational platforms facilitate students’ collaboration outside the classroom and the teachers to share teaching materials [1, 2].

Research revealed that digital photography, virtual simulations, and 3D printing in dental education improve students’ technical skills and knowledge of complicated subjects [2]. For a fast‐changing clinical environment, dental schools are integrating these technologies to ensure graduates are competent in digital workflows [3]. Pedagogy and technology need to be integrated to improve education, including AI and augmented reality [4]. YouTube is the most popular site for educational videos, but it presents notable issues regarding quality and reliability. Educational content on the platform varies in quality and depth and often lacks peer review or professional validation [5]. Research demonstrates that while many educators and learners utilize YouTube for instructional objectives, the informal approach to video creation might spread misinformation and cause misunderstandings [6]. The platform algorithm’s recommendations may prioritize interaction over educational merit, thus compromising trustworthiness [7]. However, although YouTube provides readily available knowledge, students must evaluate sources and video creators to ensure a valuable educational experience. Many factors can determine YouTube video reliability, quality, and popularity [8]. Conversely, audience engagement metrics like views, likes, dislikes, comments, and shares indicate the video’s influence rather than usefulness [9]. The purpose of this study was to evaluate the popularity, reliability, and quality of YouTube videos in dental and oral microbiology as an educational resource for dental students.

## 2. Materials and Methods

This study searched YouTube (www.youtube.com, YouTube LLC, San Bruno, CA, USA) incognito in Google Chrome on 19/09/2024 from 9:00 to 18:00. Two researchers (AH and AS) independently applied the two search terms, “dental microbiology” and “oral microbiology.” Exclusion criteria included video duration less than 2 and more than 60 min, language not English, target audience not students, duplicate videos, advertisements, or without audio. User interactions included views, likes, dislikes, comments, video length in minutes, upload date, and video authors [10].

Video popularity was calculated using the video power index (VPI) (like ratio × view ratio/100). Like ratio (like × 100/[like + dislike]) and views ratio (views/days since upload) were determined. Additionally, viewer interactions were calculated as (views/days since upload × 100%) [11]. Finally, videos were categorized by upload source: dentistry channel, dental professional, medical channel, medical professional, educational channel (nondental or medical), and for‐profit company.

Video reliability was rated using two systems. Quality Criteria for Consumer Health Information (DISCERN) was developed by Oxford University and British Library staff [12]. The second system was published by the Journal of the American Medical Association (JAMA) [13]. Subsequently, the original DISCERN was modified into a five‐item health information questionnaire (scoring: 1–5) to create mDISCERN [14]. The mDISCERN tool preserved the core concepts but added criteria for video presentations, such as visual aid clarity, presenter credibility, and content engagement. In this study, mDISCERN was further modified to assess a four‐item questionnaire. The item referring to “additional sources of information listed for patient’s reference” in the mDISCERN [14] was excluded because the audience in this study was students and not patients. Consequently, the 4 items mDISCERN with ratings from 1 to 5 give a total of 20 points for excellent reliability. Both observers’ mean values for each video were calculated and classed as 0–7 poor, 8–12 moderate, 13–15 good, and 16–20 excellent.

JAMA scoring system offers a maximum score of 4 points (authorship, attribution, disclosure, and currency). Both observers’ mean values for each video were calculated and classed as 1–2 poor, 3 good, and 4 excellent.

Video quality rating GQS is a five‐point Likert‐type scale for online video quality based on the quality, flow, and usefulness of information [15]. Two observers rated videos, and the mean scores from both observers were rated as poor (1–2), moderate (3), good (4), and excellent (5).

### 2.1. Statistical Analysis

The data were analyzed using the Statistical Package for Social Science (SPSS), IBM SPSS Statistics v26. The Shapiro–Wilk test was used to evaluate the normality of the data. ANOVA test was to compare means of the variables between the two observers. Cohen’s Kappa was applied to compare inter‐ and intraobserver agreement. Intergroup comparisons were carried out using nonparametric Kruskal–Wallis test or parametric 1‐way analysis of variance according to data distribution. A *p*‐value of 0.05% and 95% confidence interval (CI) were considered the significance level.

## 3. Results

**Figure 1:**
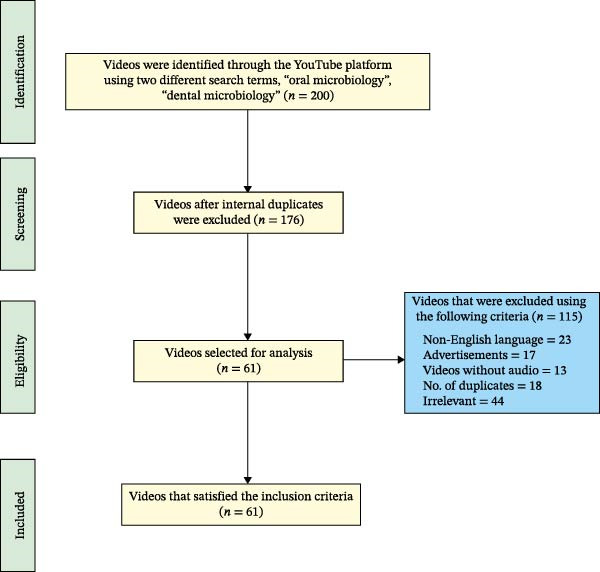
Summary of the search strategy with exclusion criteria and workflow diagram.

YouTube searches into the two phrases yielded 200 videos, of which 139 had been excluded based on the established criteria (Figure 1). The inter‐ and intraobserver agreements between the two observers for video variables were significant, with values ranging from 0.943 to 0.9. The mean values of each variable were computed from the two observers and used for analysis. The Shapiro–Wilk test revealed a significant deviation from normality in the distribution of the data (W122) = 0.22–0.863, *p* = 0.001.

Table 1 presents the characteristics of the analyzed videos, noting that no video received a dislike, and thus dislikes were given zero in all calculations. The video that generated the highest views achieved 2,830,989 views and 27,000 likes and was uploaded for 2598 days before the variable scoring. The most recent video was uploaded 95 days ago, whereas the oldest was published 3494 days before the study commenced. The videos have a duration that varies from a minimum of 3:32 min to a maximum of 56:33 min. The count of comments ranged from 0 to 610, while the dislike count was zero for all analyzed videos.

**Table 1:** Characteristics of videos.

Variables	Median (min–max)
Days since the video was uploaded	1063 (95–3494)
Video duration (min)	15:13 (3:32–56:33)
Views count	3123 (1–2,830,989)
Views ratio	3.17 (0–1089.68)
Viewing rate	20.41 (0.5–20,776.12)
Likes count	56 (0–27,000)
Likes ratio	1.79 (0–1044.41)
Video power index	0.06 (0–9227.47)
Comments count	2 (0–610)

Table 2 shows that out of the 61 selected videos, the search terms “dental microbiology” and “oral microbiology” resulted in 44 and 17 videos, respectively. Nonetheless, the search term “oral microbiology” achieved the highest statistically significant median views (8684) and like counts (201) (*p*  < 0.05), despite a median video length of 12:25 min (*p*  < 0.05).

**Table 2:** Comparisons of videos based on the search term.

Variables	Dental microbiology (*n* = 44)	Oral microbiology (*n* = 17)	*p*‐Value
	Median (min–max)	Median (min–max)	
Days since upload	847 (195–3994)	1496 (95–2598)	0.137
Video duration	19:43 (4:28–56:32)	12:25 (3:32–39:34)	0.045 ^∗^
Views count	1515 (1–391,192)	8684 (626–27,000)	0.005 ^∗^
Likes count	16 (0–4700)	201 (0–27,000)	0.011 ^∗^
Comments count	0.5 (0–456)	9 (0–610)	0.175

*Note:* Kruskal–Wallis test.

^∗^ *p*‐value < 0.05.

Table 3 presents an analysis of video duration in minutes, indicating that videos between 2 and 10 min received significant popularity, evidenced by the VPI (*p*‐value < 0.05). In contrast, videos exceeding 30 min received the lowest VPI (*p*‐value < 0.05).

**Table 3:** Analysis of variables based on video duration in minutes.

Variables	2–10 min (*n* = 15)	11–30 min (*n* = 29)	31–60 min (*n* = 17)	*p*‐Value
	Median (min–max)	Median (min–max)	Median (min–max)	
Views	8347 (55–2,830,989)	8612 (1–75,534)	115 (15–69,534)	0.009 ^∗^
Views ratio	3.22 (0.28–1089.68)	6.75 (0–499.89)	0.15 (0.02–49.45)	0.020 ^∗^
Viewing rate	41.94 (0.05–20,776.07)	33.08 (0.07–19,017.6)	4.31 (0.25–4945.48)	0.705
Likes	96 (0–27,000)	147 (0–14,500)	2 (0–1800)	0.029 ^∗^
Likes ratio	1.59 (0–1074.44)	2.02 (0–959.63)	1.73 (0–25.65)	0.359
Video power index	0.14 (0–9227.47)	0.02 (0–4801.8)	0.003 (0–2.96)	0.038 ^∗^
Comments count	3 (0–610)	5 (0–329)	0 (0–330)	0.430
mDISCERN	11 (0–15)	12 (0–16)	11.5 (10–14)	0.259
JAMA	3 (1–4)	3 (1–4)	2 (2–3)	0.061
GQS	3 (2–5)	3 (2–5)	3 (3–4)	0.360

*Note:* Kruskal–Wallis test.

^∗^ *p*‐value < 0.05.

Table 4 shows the differences among the sources of videos uploaded to YouTube across all variables. The sources of upload were classified into several categories: dental channel, dental professional, medical channel, medical professional, educational channel, and for‐profit company. Most variables showed statistically significant differences across categories (*p*‐value < 0.05), except for viewing rate and GQS. Among the 61 videos analyzed, the majority were uploaded by dentistry channels (*n* = 26), with dental professionals following at a count of 13. Educational channels uploaded videos that achieved the highest median daily views of 274, while for‐profit companies such as publishers received the second‐highest daily views of 36. However, dental professionals received a median of 8 views per day. In contrast, dental channels received the lowest daily viewership of 0.07 (*p*‐value < 0.05). Educational channels with a median VPI of 2776 uploaded the most popular videos, whereas dental channels were the least popular with a median VPI of 0.002.

**Table 4:** Comparisons of videos based on the source of upload.

Variables	Dent chanel (*n* = 26)	Dent prof (*n* = 13)	Med chanel (*n* = 8)	Med prof (*n* = 6)	Ed chanel (*n* = 2)	Company (*n* = 6)	*p*‐Value
	Median (min–max)	Median (min–max)	Median (min–max)	Median (min–max)	Median (min–max)	Median (min–max)	
Views	62 (1–156,758)	11,084 (821–137,443)	52,591 (2251–755,341)	2362 (55–9366)	64,618 (3295–1,288,941)	54,451 (2661–2,830,989)	0.001 ^∗^
Views ratio	0.07 (0–7211)	8.33 (0.88–69.7)	28.27 (2.38–499.89)	2.5 (0.28–4.09)	273.8 (34.68–51,291)	36.12 (1.14–1089.68)	0.001 ^∗^
Viewing rate	4.63 (0.07–7710.65)	601.64 (0.5–6969.73)	17.91 (0.25–20,776.12)	129.91 (0.06–319.18)	28.46 (20.41–36.51)	81.23 (0.05–19,017.6)	0.141
Likes	2 (0–2500)	242 (11–1800)	920 (37–14,500)	35 (2–100)	13,556 (112–27,000)	973 (0–22,000)	0.001 ^∗^
Likes ratio	1.62 (90–134.27)	2.18 (0.89–23.64)	14.78 (1.0–959.63)	1.79 (1.03–4.26)	569.15 (117.89–1074.41)	1.49 (0–846.81)	0.011 ^∗^
Video power index	0.002 (0–50.14)	0.26 (0.01–2.59)	2.52 (0.09–4801.8)	0.033 (0.01–0.14)	2775.83 (4.89–5510.76)	1.46 (0–9227.47)	0.001 ^∗^
Comments count	0 (0–158)	10 (0–70)	20 (0–456)	2 (0–330)	309 (9–610)	17 (0–250)	0.002 ^∗^
mDISCERN	11.5 (0–16)	12.5 (11–14)	10.5 (6–14)	10.25 (7–11)	13.75 (13–15)	13 (0–15)	0.009 ^∗^
JAMA	2 (1–4)	3 (2–4)	2.5 (2–3)	2.25 (1–4)	3 (3–3)	3.75 (1–4)	0.012
GQS	2.75 (2–4)	3 (3–4)	3 (3–4)	3 (2–4)	3.5 (3–5)	3 (2–5)	0.336

*Note:* Kruskal–Wallis test.

^∗^ *p*‐value < 0.05.

Table 5 displays the first reliability assessment of videos, organized according to the median mDISCERN scores, which are classified into poor, moderate, good, and excellent categories. Statistical significance was observed in only three variables: viewing rate, comments count, and GOS (*p*‐value < 0.05). Out of the 61 videos examined, only two demonstrated excellent reliability, while 21 exhibited good reliability, with statistically significant GQS quality (*p*‐value < 0.05).

**Table 5:** Comparison of videos based on mDISCERN score.

Variables	Poor (*n* = 8)	Moderate (*n* = 30)	Good (*n* = 21)	Excellent (*n* = 2)	*p*‐Value
	Median (min–max)	Median (min–max)	Median (min–max)	Median (min–max)	
Views	2666 (67–35,649)	1271 (1–2,830,989)	8684 (27–391,192)	92,969 (29,181–156,758)	0.172
Views ratio	2.22 (0.04–20.17)	0.94 (0–1089.68)	6.57 (0.03–19.18)	57.21 (37.32–77.11)	0.112
Viewing rate	1.94 (0.05–488.13)	16.39 (0.06–20,776.12)	134.97 (0.50–19,017.6)	3857.71 (4.77–7710.65)	0.041 ^∗^
Likes	29 (0–540)	17 (0–27,000)	173 (0–4700)	1775 (1050–2500)	0.142
Likes ratio	3.72 (0–30.56)	1.68 (0–1074.41)	1.83 (0–117.89)	67.93 (1.59–134.27)	0.562
Video power index	0.08 (0–6.17)	0.12 (0–9227.47)	0.20 (0–40.89)	25.66 (1.23–50.14)	0.169
Comments count	0 (0–15)	0 (0–610)	8 (0–250)	115 (72–158)	0.029 ^∗^
JAMA	2.5 (1–4)	2 (1–4)	3 (2–4)	3 (3–3)	0.111
GQS	2.5 (2–4)	—	3 (3–5)	3.5 (3–4)	0.011 ^∗^

*Note:* Kruskal–Wallis test.

^∗^ *p*‐value < 0.05.

Table 6 presents the second reliability tool, JAMA scores, with the results classified into poor, good, and excellent categories. Median JAMA scores revealed significant differences (*p*‐value < 0.05) across all variables, with the exception of viewing rate, comment counts, and mDISCERN. Among the 61 videos evaluated, six demonstrated excellent and 22 good reliability with statistically significant quality (GQS) (*p*‐value < 0.05).

**Table 6:** Comparison of videos based on JAMA score.

Variables	Poor (*n* = 33)	Good (*n* = 22)	Excellent (*n* = 6)	*p*‐Value
	Median (min–max)	Median (min–max)	Median (min–max)	
Views	135 (1.0–1,288,941)	6114 (55–755,341)	33,168 (1582–2,830,989)	0.002 ^∗^
Views ratio	0.77 (0–512.91)	6.42 (0.28–499.89)	23.81 (1.0–1089.68)	0.002 ^∗^
Viewing rate	7.51 (0.05–20,776.12)	122.55 (0.1–7710.65)	1.75 (0.06–1917.6)	0.199
Likes	2 (0–27,000)	143 (2–14,500)	614 (23–22,000)	0.001 ^∗^
Likes ratio	1.65 (0–1074.41)	2.41 (0.89–959.63)	13.95 (1.2–846.81)	0.007 ^∗^
Video power index	0 (0–5510.76)	0.17 (0.01–4801.8)	2.44 (0.01–9227.47)	0.001 ^∗^
Comments count	0 (0–610)	2.5 (0–330)	7 (0–250)	0.065
mDISCERN	11.5 (0–16)	12 (0–15)	13 (11–15)	0.132
GQS	2.5 (2–4)	3 (2–5)	3 (3–5)	0.009 ^∗^

*Note:* Kruskal–Wallis test.

^∗^ *p*‐value < 0.05.

Table 7 presents a comparison of video quality using GQS median scores, categorizing them as poor, moderate, good, and excellent. The median GQS shows notable differences (*p*  < 0.05) across all variables, with the exception of the likes ratio and JAMA. Out of the 61 videos assessed, only four demonstrated excellent quality, achieving the highest median counts across all variables, including reliability rating mDISCERN, with the exception of comment counts. Furthermore, 10 videos demonstrated significantly good quality.

**Table 7:** Comparison of videos based on GQS score.

Variables	Poor (*n* = 10)	Moderate (*n* = 37)	Good (*n* = 10)	Excellent 5 (*n* = 4)	*p*‐Value
	Median (min–max)	Median (min–max)	Median (min–max)	Median (min–max)	
Views	2642 (67–1,288,941)	1582 (1–2,830,989)	14,829 (55–310,603)	28,780 (3295–391,192)	0.020 ^∗^
Views ratio	9.26 (0.04–512.91)	1.0 (0–1089.68)	13.22 (0.28–207.76)	35.12 (4.65–190.18)	0.008 ^∗^
Viewing rate	12.45 (0.05–1837.72)	4.77 (0.06–1219.66)	1321.58 (27.95–20,776.12)	2010.27 (36.51–19,017.6)	0.001 ^∗^
Likes	90 (0–27,000)	9 (0–22,000)	412 (72–4700)	539 (72–4700)	0.017 ^∗^
Likes ratio	2.46 (0–1074.41)	1.73 (0–959.63)	1.94 (1.0–3.68)	1.9 (1.2– 117.89)	0.700
Video power index	0.2 (0–5510.76)	0.01 (0–9227.47)	0.30 (0.01–2.07)	1.46 (0.09–4.89)	0.041 ^∗^
Comments count	2.5 (0–610)	0 (0–330)	22 (0–456)	21 (4–250)	0.0423 ^∗^
mDISCERN	10 (0–15)	11.5 (6–16)	12.5 (10–15)	13.25 (13–14)	0.040 ^∗^
JAMA	2.75 (1–4)	2 (1–4)	3 (2–3)	3.25 (3–4)	0.067

*Note:* Kruskal–Wallis test.

^∗^ *p*‐value < 0.05.

## 4. Discussion

This study evaluated the popularity, reliability, and quality of YouTube videos using VPI for popularity, JAMA and mDISCERN for reliability, and GQS for quality. YouTube videos complement lectures and improve student engagement and knowledge in blended learning. Recent research emphasizes the value of using video content in education to offer students personalized learning experiences and allow them to review complex topics [16].

We selected “dental microbiology” and “oral microbiology,” search terms that students likely use to search relevant videos that include bacteria, viruses, fungi, and other microorganisms associated with oral disease. Our results showed significant differences between the two search terms. The search term “oral microbiology” had the most median views and likes, showing that it appeals to a wider audience due to public knowledge of the oral microbiome’s role in systemic health. Furthermore, the high popularity metrics in oral microbiology searches indicate that content creators are reaching and engaging with wider audience [17].

Video popularity depends on VPI and video characteristics. While not a direct measure of quality or reliability, VPI influences video’s popularity, engagement, reach, and viewer retention. In this study, the median VPI is 0.06, compared to a VPI of 0.11 [18], and a second VPI showed 0.09 [19]. The latter finding matches ours but suggests more popularity. However, our findings may help dental education video developers to aim for a greater VPI to improve viewer popularity.

We selected video lengths between 3 and 60 min since earlier studies reported that videos under 3 min were insufficient and incomplete [20, 21]. Conversely, videos longer than 60 min frequently struggle to engage viewers, reducing attention spans and retention [22]. Our results showed that most videos (47.5%) are between 11 and 30 min long but less popular than those under 10 min. Educational channels targeting medical and dental students had the most daily views followed by for‐profit companies. However, dental professionals and dentistry channels had the least daily views. The latter groups posted videos longer than 30 min, some of which are direct recordings of face‐to‐face lectures. Despite their educational content and intellectual merit, these videos have little views and likes. This finding supports research that demonstrated YouTube’s algorithm prioritizes visually appealing content over academic rigor [23].

Since JAMA and mDISCERN are popular evaluation systems, we employed both tools to measure video reliability. Our data show that 9.8% of the videos have excellent JAMA reliability ratings, with 36% good. Similarly, mDISCERN revealed 3.3% of videos were excellent and 34.4% good. These findings are concerning since they show that most of the assessed videos are unreliable despite being produced by educational channels and professionals. Our findings are consistent with research that critically analyzed video content as a source of health education and found that many do not meet reliability standards [24]. The disparities between JAMA and mDISCERN emphasize the importance of using precise evaluative systems to ensure students receive accurate and reliable instructional material.

We evaluated the videos’ quality using the GQS. We identified 6.6% excellent and 16.4% good videos. The low percentage of excellent videos may be a call for educational video publishers to make improvements. This statement supports the view that digital educational content is becoming more competitive, requiring educators to emphasize quality to and improve educational usefulness [25]. A study of 55 COVID‐19 prevention videos for dentists found 3.2% of good quality [26]. Gupta et al. [27] analyzed 92 video on tooth fracture management from YouTube and Vimeo, showing that videos produced by dental professionals exhibited significantly higher DISCERN and JAMA scores; yet, the overall quality of the videos remains unsatisfactory. Furthermore, Akcay et al. [28] analyzed 48 YouTube videos on computer‐controlled local anesthesia in dentistry and showed that videos from academic sources exhibited significantly higher quality and reliability scores. However, viewer engagement metrics are insufficient as reliable indicators of educational value, as lower quality content may still achieve popularity. These studies emphasize the significance of employing many evaluation criteria to achieve a comprehensive understanding of video quality, which is necessary for educators and students in search of reliable online materials. Future research may examine the influence of viewership metrics and algorithms on the quality of educational videos through longitudinal studies.

The limitations of this study result from the dynamic nature of YouTube video uploads, where publishers can delete or upload videos at any time. The study results may vary depending on the date and time of the search. Two search terms were used; thus, the study results may vary depending on the chosen search terms. Evaluating only English‐language videos might hide useful content made in other languages.

## 5. Conclusion

In this study, we found that YouTube algorithm favors shorter videos for audience engagement over academic rigor. Most videos were not educationally useful because reliability and quality were not addressed throughout production. Educators should scientifically create and write high‐quality videos. This can be achieved by applying multiple reliability and quality tools to enhance understanding and learning experience.

## Funding

This study received no funding.

## Ethics Statement

This is observational research based on publicly available data. No ethical approval is needed.

## Conflicts of Interest

The authors declare no conflicts of interest.

## Data Availability

Data are available on request from the authors.
